# Sorption of aspartic and glutamic aminoacids on calcined hydrotalcite

**DOI:** 10.1186/2193-1801-2-211

**Published:** 2013-05-08

**Authors:** Fabiano Silvério, Márcio José dos Reis, Jairo Tronto, João Barros Valim

**Affiliations:** Departamento de Química - Faculdade de Filosofia Ciências e Letras de Ribeirão Preto, Universidade de São Paulo, Av. Bandeirantes, 3900, CEP 14040-901 Ribeirão Preto, Brazil; Universidade de Franca, Av. Dr. Armando Salles Oliveira, 201, Parque Universitário, CEP14404-600 Franca, SP Brazil

**Keywords:** Hydrotalcite, Layered double hydroxides, Aminoacids, Adsorption, Sorption

## Abstract

Sorption of aspartic and glutamic aminoacids by regeneration of calcined hydrotalcite is reported. Hydrotalcite was synthesized by coprecipitation and calcined at 773 K. Sorption experiments were performed at 298 K and 310 K, and the results reveal that at low aminoacids equilibrium concentrations, intercalation of hydroxyl anions takes place while at high equilibrium concentrations, the sorption process occur by means re-hydration and aminoacids intercalation of hydrotalcite. The results also suggested that Asp and Glu sorption is a temperature dependent process. The amount of sorbed amino acid decreases as the temperature increase. The effect is more pronounced for Glu sorption probably due to its higher hydrophobic character, which makes the sorption more difficult in comparison with sorption of Asp at higher temperature.

## Introduction

Hydrotalcite-like compounds, also known as Layered Double Hydroxides (LDH) have received considerable attention due to their properties and applications (Costantino et al. 2008; Takehira & Shishido 2007; Darder et al. 2007; Evans & Xue 2006; Velu et al. 2005; Anbarasan et al. 2005; Zhu et al. 2005; Tronto et al. 2004). Their structure consists of sheets disposed in a layered array formed by octahedral sharing their edges, with bi and trivalent cations on the centers of octahedral hexacoordinated with hydroxyl anions. Layers of LDH are residual positive charge neutralized by anions located in the interlayer domain.

Hydrotalcite can be used to remove anions from aqueous solution by three different processes: adsorption, anion exchange and regeneration of a calcined precursor (Zhu et al. 2005; Takehira et al. 2005; Aisawa et al. 2004). Mg-Al and Zn-Al LDH systems present the specific property known “memory effect” that consists in the capacity of calcined LDH regenerate its lamellar structure by incorporation of anions when it is put in contact with an intercalating anion in aqueous solution (Kooli et al. 1997). Taking advantage of the “memory effect”, different molecules such as polyorganic anions, benzoate, tereftalate and surfactants, have been sorbed onto LDH (Cardoso et al. 2003; Cardoso et al. 2004; Crepaldi et al. 2002).

Aspartic (Asp) and glutamic (Glu) aminoacids are used in pharmaceutical and food industry, where industrial wastewater treatment is not often practiced (Ohtsubo et al. 2005; Shih & Van 2001). These aminoacids differ due to an extra CH_2_ group in the Glu aliphatic chain and both have a carboxylic group. This work is focused on evaluation of sorption process of Asp and Glu aminoacids by regeneration of calcined MgAl-LDH in order to verify the efficiency of its adsorbent for wastewaters treatment.

## Materials and methods

### Layered double hydroxide – the sorbent

The LDH was prepared by coprecipitation at variable pH as proposed by Reichle (Reichle et al. 1986). All reactants were of analytical grade and were used without further purification. Magnesium Nitrate (>99%), Aluminum Nitrate (>98%), Sodium Hydroxide (>98%) and Sodium Carbonate (>99%) were purchased from Merck. All solids were characterized by Powder X-Ray Diffraction (PXRD), using a Siemens D5005 X-ray Diffractometer, with a graphite monochromator selecting the Cu-Kα_1_ radiation (0.15406 nm) in an angular 2θ range of 2-70° and step rate of 0.02° s^-1^; Fourier Transform Infra-Red Spectroscopy (FT-IR), with an ABB Bomem MB 100 spectrometer over the range 400–4000 cm^-1^ with 32 scans and a 4 cm^-1^ resolution, using pressed KBr pellets at 2% (w/w) of sample; Thermogravimetric and Differential Thermal Analysis (TGA/DTA), using a TA Instruments SDT 2960 in synthetic air atmosphere at a heating rate of 10 K min^-1^; Scanning Electron Microscopy (SEM) using a Zeiss DSM 960-Digital Scanning Microscope; and Specific Surface Area (SSA) performed on Quanta Chrome Nova 1200 equipment.

From TGA/DTA and elemental analysis the formula of the LDH precursor was obtained as , which corresponds to a Mg/Al ratio of 2.3/1. This information is extremely important because the anionic exchange by regeneration depends on of this ratio. Immediately before use in adsorption, the LDH precursor was calcined at 773 K for 4 hours under O_2_ (White Martins) flow giving rise a Mg-Al mixed oxy-hydroxide – the adsorbent.

### Aspartic and glutamic acids – the sorbates

The aminoacids (Asp and Glu) were acquired from Merck (>99.5% assay), and used without further purification. All aminoacids solutions were prepared with deionized water (MilliQ®), and the pH was adjusted to 10 with NaOH.

### Adsorption/sorption experiments

Sorption experiments of Asp and Glu were carried out in bath method with 100 mg of the calcined precursor into 25 cm^3^ of amino acid solutions at different concentrations (concentration ranging from 0.001 to 0.04 mol.dm^-3^ for Asp, and from 0.001 to 0.06 mol.dm^-3^ for Glu) at pH 10. The obtained suspension was ultra-sonicated for 10 minutes to homogenize particle size, before adsorption. The isotherms were obtained at 298 K and 310 K.

Closed samples were place in a thermostatic bath with orbital shaking, for 70 hours, to ensure that the sorption equilibrium would be reached. After that, each sample was divided into two parts: one was centrifuged at 10,000 G for 20 minutes and the supernatant was used to quantifier the amount of amino acid, using a UV–vis 8453 Hewlett Packard spectrophotometer, and the solid was dried and characterized by PXRD and FTIR; the other part of the sample was kept in aqueous suspension, during 10 minutes, until largest particles were decanted, and after that, it was used for determination of electrokinetical (zeta) potential. The measurement of electrokinetical potential was carried out in triplicate at the same temperature of sorption experiments.

## Results and discussion

**Figure 1:**
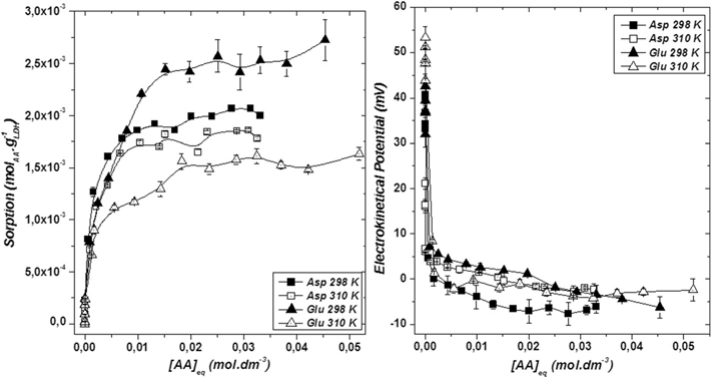
**Left: Isotherms; Right: Electrokinetical potential related to the aminoacids sorption.**

In Figure 1 presents the sorption isotherms obtained for Asp and Glu. Both isotherms indicate that an increase in temperature of the system results in lower amounts of amino acid removed. For Asp sorption, the maximum amount removed was 2.0 × 10^-3^ mol.g^-1^ and 1.8 × 10^-3^ mol.g^-1^ at 298 K and 310 K respectively, while for Glu the maximum amount removed was 2.7 × 10^-3^ mol.g^-1^ and 1.6 × 10^-3^ mol.g^-1^.

The lower amount of amino acid removed obtained at 310 K can be explained considering that with at higher temperature of the system, the higher is the importance of entropy for the system’s Gibb’s free energy (ΔG = ΔH – TΔS), thus the role of enthalpy is reduced. Thus, organization of compact aggregates with a larger number of amino acid molecules at the LDH should become more difficult. Moreover, as the experiments were performed in aqueous medium, the interlayer section of the LDH provides an environment energetically most suitable to host hydrophobic molecules. At 298 K this effect is intensified in favor of Glu intercalation due to its higher hydrophobic character than Asp. Therefore, the more hydrophobic is the amino acid, the more it will be sorbed at the same equilibrium concentration at 298 K while the opposite trend occurs at 310 K.

At lower aminoacids concentrations, the sorption process does not seem to be influenced by temperature and the amounts of aminoacids removed are approximately the same for all conditions. The LDH reconstruction with both aminoacids seems to be very similar. The regeneration at low aminoacids concentrations occurs predominantly via intercalation of OH^-^ anions from aqueous solution (pH 10). As the amino acid concentration increases, a competition between OH^-^ and amino acid takes place with amino acid intercalation. At lower amino acid concentrations, approximately 99% of Asp or Glu are removed; whereas, at higher amino acid concentration, near the limit of solubility, the extraction rate is approximately 20%. The electrokinetical potential curves related to each isotherm are also very similar. Positives values at low equilibrium concentrations decrease, reaching values as negative as −6 mV, while the aminoacids concentration increase. The profiles of electrokinetical potential curves are in agreement with the respective isotherm profile.

The amount of charge available for removal of anionic species by the calcined LDH was calculated taking into account the amount of Al^3+^, and it was found 5.36 × 10^-3^ mol of charge (+1) per g of calcined LDH. Then, 2.68 × 10^-3^ mol of Asp or Glu could be removed by anionic exchange. The maximum amount remove (after isotherms) is about 2.7 × 10^-3^ mol Glu per g of LDH at 298 K. The other values in the corresponding isotherms are lower.

**Figure 2:**
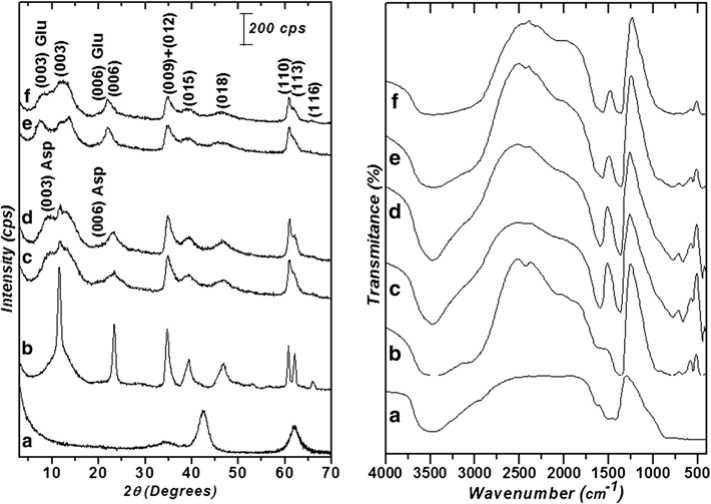
**Left: PXRD; Right: FTIR. a**) calcined LDH; **b**) LDH regenerated with OH^-^ anions; **c**) LDH sorbed with Asp (298 K); **d**) LDH sorbed with Asp (310 K); **e**) LDH sorbed with Glu (298 K); **f**) LDH sorbed with Glu (310 K).

In Figure 2 are presented results obtained by PXRD and FTIR for the calcined LDH and the solids obtained after sorption experiments. For the calcined LDH, the diffractogram did not give evidence of a lamellar structure, but only peaks assigned to the mixed oxide could be seen. On the other hand, the LDH regenerated at pH 10 presented a basal spacing of 7.4 Å, which suggests intercalation of OH^-^ anions. The solids regenerated at higher Asp and Glu concentrations presented a basal spacing of 11.4 Å and 12.2 Å, respectively, calculated by Bragg equation. Considering the LDH layer width of 4.8 Å in addition to the hydrogen bonding space, interlamellar spacing of 6.6 Å and 7.4 Å are then obtained for the interleaved Asp and Glu. This suggests that intercalation occurs with the amino acids aligned parallel to each other and perpendicular to the layers of the LDH (Tronto et al. 2003) although two series of peaks 00l indicating the presence of other anion besides the aminoacids. The FTIR spectra obtained for calcined LDH are characterized by an intense broad band at 3400 cm^-1^, which is due to O-H stretching of the hydroxyl groups and water. The presence of aminoacids can be evidenced by two bands at 1590 and 1400 cm^-1^ related to the asymmetric and symmetric stretching of carboxylate group (Fudala et al. 1999; Aisawa et al. 2002; Whilton et al. 1997).

The thickness of the platelets of the materials was also calculated from PXRD using Debye-Scherer method (West 1987). The results presented in Table 1 suggest good re-stacking of the LDH in water and at low Asp and Glu concentrations. On the other hand, the solids obtained at higher aminoacids concentrations (in the plateau of sorption) presented lower structural organization. Specific surface area (SSA) was determined (Table 1) for all materials are lower than that determined for the calcined material.

**Table 1:** **General parameters observed for the material sorbed with Asp and Glu at 298 K and 310 K**

Sample	Medium particle size (Å)	SSS (m^2^.g^-1^)	Pore total volume (cm^3^.g^-1^)	Average pore diameter (Å)
LDH calcined	--	191.4	0.250	52.3
Regenerated with OH	251.9	47.6	0.284	238.8
Initial area (Asp-298 K)	248.4	51.5	0.282	219.2
Initial area (Glu-298 K)	320.3	57.5	0.284	196.7
Last point (Asp-298 K)	48.3	1.7	0.010	238.9
Last point (Asp-310 K)	85.5	6.7	0.021	224.6
Last point (Glu-298 K)	88.3	1.6	0.006	151.2
Last point (Glu-310 K)	88.2	2.2	0.009	172.9

**Figure 3:**
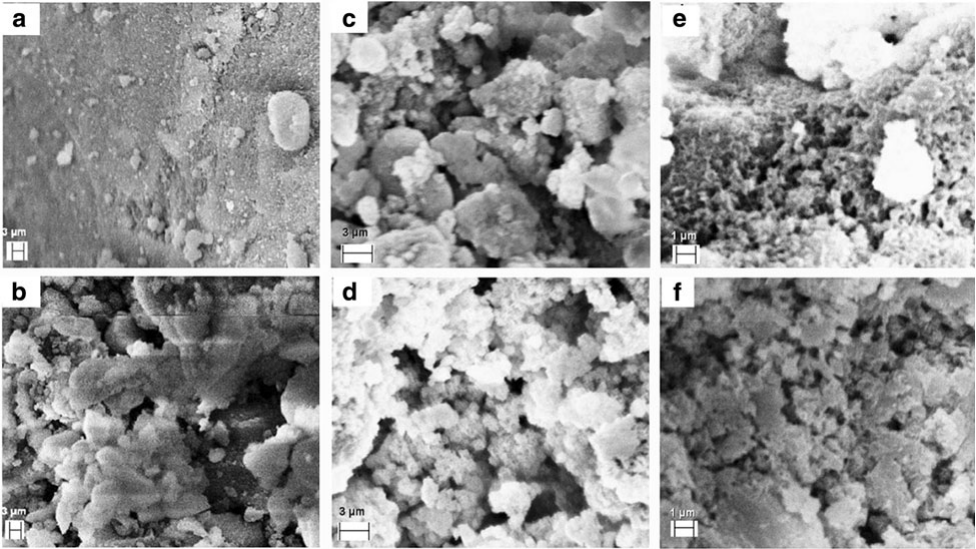
**SEM images of the LDH under different conditions. a**) coprecipitated LDH; **b**) calcined LDH; **c**) LDH sorbed with Asp (298 K); **d**) LDH sorbed with Asp (310 K); **e**) LDH sorbed with Glu (298 K); **f**) LDH sorbed with Glu (310 K).

SEM images obtained for the LDH before and after aminoacids sorption is presented in Figure 3. The MgAl-CO_3_-LDH precursor presents irregular surface with low pororosity and few aggregates is observed (Figure 3a) while the calcined LDH (Figure 3b) presents high porous surface, in agreement with SSA. The images obtained for the material sorbed with Asp (Figure 3c and d) and Glu (Figure 3e and f) present white aggregates in surface due to the presence of less conductive organic species. These materials are less porous which also agrees with SSA.

## Conclusions

The results showed that Asp and Glu intercalation by sorption is a process that dependent on the amino acid concentration. At low amino acid concentrations, the LDH is regenerated predominantly with intercalated hydroxyl anions. As the amino acid concentration increases, a competition between the hydroxyl anions and the amino acid for intercalation in the interlayer domain takes place with amino acid dislocating the equilibrium in favor of Asp or Glu intercalation. The results also suggested that Asp and Glu sorption is a temperature dependent process, with a decrease in the amount of sorbed amino acid with increasing temperature. This effect is more pronounced in the case of Glu, probably due to its higher hydrophobic character, which makes the sorption more difficult in comparison with sorption of Asp at higher temperature. Thus, aminoacids hydrophobicity contributes to sorption: the more hydrophobic is the amino acid, the more it will be sorbed at the same equilibrium concentration at 298 K, while the opposite trend occurs at 310 K.
